# Special Issue “Equine Viruses”: Old “Friends” and New Foes?

**DOI:** 10.3390/v12020153

**Published:** 2020-01-29

**Authors:** Romain Paillot

**Affiliations:** 1 LABÉO, Equine Health Department, Research Division, 14280 Saint-Contest, France; romain.paillot@laboratoire-labeo.fr; Tel.: +33-2-3147-1926; 2 NORMANDIE UNIV, UNICAEN, BIOTARGEN, 14000 Caen, France

The Food and Agriculture Organization of the United Nations has recently estimated that the world equid population exceeds 110 million (FAOSTAT 2017). Working equids (horses, ponies, donkeys, and mules) remain essential to ensure the livelihood of poor communities around the world. In many developed countries, the equine industry has a significant economical weight, with around 7 million horses in Europe alone. The close relationship between humans and equids, and the fact that the athlete horse is the terrestrial mammal that travels the most worldwide after humans, are important elements to consider in the transmission of pathogens and diseases, amongst equids and to other species. The potential effect of climate change on vector ecology and vector-borne diseases is also of concern for both human and animal health.

With this Special Issue, which assembles a collection of communications, research articles, and reviews, we intend to explore our understanding of a panel of equine viruses, looking at their pathogenicity, their importance in terms of welfare and potential association with diseases, their economic importance and impact on performance, and how their identification can be helped by new technologies and methods. Beyond their potential risk to other species, including humans, equine viruses may also represent an interesting model for reproducing virus infection in the host species.

Dennis et al. [1] contributed a review on African Horse Sickness (AHS). This disease, caused by the *orbivirus* AHS virus (AHSV), induces a very high mortality rate that can exceed 95% in its most severe form. This disease mostly occurs in southern African countries, but its transmission by *Culicoides* biting midges is of great concern in the current context of global warming and its consequence on displacement of vector populations. In the absence of treatment, prevention is essential, and Dennis et al. also provide a comprehensive review of the different vaccine strategies and technologies available and in current development against AHSV. While live attenuated and inactivated AHSV vaccines have played a role to reduce the impact and occurrence of AHS in affected areas, the number of AHSV serotypes in circulation and their lack of DIVA markers (differentiating infected from vaccinated animals) is a drawback that leads to the development of a new generation of vaccines, such as poxvirus-vectored or reverse genetics vaccines. Lecollinet et al. [2] reviewed major viruses inducing encephalitis in equids and their growing importance as a threat to the European horse population. Amongst them, equid herpesviruses (EHVs) are some of the most frequently isolated equine viruses worldwide. The equine herpesvirus type 1 (EHV-1) is of particular interest to the equine industry because of the different forms of disease it can induce, from a mild respiratory infection to abortion, neonatal death, and myeloencephalopathy (EHM) [3]. A communication from Preziuso et al. [4] and an article from Sutton et al. [5] specifically focused on EHV-1 strain characterization in order to better understand EHV circulation in Italy and France. Different approaches were compared, from the single-nucleotide point (SNP) mutation in ORF30 (historically associated with abortive or neuro-pathogenic strains), to other ORF gene sequences and the newly described multilocus strain typing methods (MLST; [6]). The MLST method is an interesting new approach for EHV-1 and a potential epidemiological tool that could provide an alternative until the development of more accessible EHV whole-genome sequencing methods. EHV-1 strain characterization by Sutton et al. allowed to conclude that the surge of EHV-1 outbreaks reported in France in 2018 was not linked to the introduction and/or circulation of a new EHV-1 strain in the French horse population. The origin of this crisis could be linked to a shortage of EHV vaccine and a subsequent reduced rate of EHV vaccination in the preceding years [7]. Lecollinet et al. also reviewed less frequently isolated encephalitis viruses, which may be zoonotic, such as rabies virus, borna disease virus, and West Nile virus (WNV). In the case of WNV, both horses and humans are highly susceptible to viral infection through infected mosquitoes. Unprecedented circulations of WNV have been observed in several European countries in the last decade, with a potential role of climatic and environmental conditions. Both species are considered as dead-end hosts. However, the horse could be used as a sentinel species to monitor and control vector-borne virus activity. Other enzootic flaviviruses were also reviewed, such as Tick-Borne Encephalitis virus (TBEV) and Louping Ill virus (LIV). In Europe, vaccination is only available against some of these pathogens (i.e., EHV-1 and 4, Rabies virus, and WNV), which highlights the importance of surveillance. Taking into account that most of these viruses will induce similar clinical signs of disease, the development of discriminative diagnostic tools is also of increasing importance. Finally, the review presents some other vector-borne (mosquitoes or midges) equine encephalitis viruses, not currently circulating in Europe, from the *Flaviridae* family (i.e., the Japanese encephalitis virus (JEV), Saint Louis encephalitis virus (SLEV), and Murray Valley encephalitis virus (MVEV)) or the alphaviruses from the *Togaviridae* family (i.e., eastern, western, and Venezuelan equine encephalitis viruses; EEEV, WEEV, and VEEV, respectively). In relation to VEEV, Rusnak et al. [8] presented systematic approaches for strain selection and propagation of virus and challenge material for the development and approval of a VEEV vaccine under the FDA Animal Rule and the different animal models available (rodents and non-human primates).

Altan et al. [9] have used metagenomics to identify viruses in horses with neurological and respiratory diseases. The equine hepacivirus (EqHV) was detected in the plasma from several neurological cases. This virus, which was first reported in horses in 2012 [10], was further investigated by Badenhorst et al. [11], with a specific focus on its circulation in Austria and the potential role of mosquitoes in its transmission. The prevalence of EqHV in the Austrian horse population studies reached 45% (based on serological evidence), with around 4% of samples positive for EqHV RNA. No EqHV RNA was found in mosquitoes collected across Austria, raising questions about its methods of transmission. Some aspects of this particular question of EqHV transmission were treated by Pronost et al. [12], who presented evidence to support a potential in utero transmission of EqHV from the mare to the foal, based on three positive clinical cases amongst 394 dead foals screened for the presence of EqHV RNA (prevalence of 0.76%). Altan et al. [9] also detected two copiparvoviruses, the equine parvovirus-hepatitis (EqPV-H) and a new one named *Eqcopivirus* by the authors, with no specific and/or statistical association with disease. Equine parvovirus-hepatitis was also the subject of the article from Meister et al. [13], which reported an EqPV-H infection occurrence in a quarter of the actively breeding Thoroughbred horse population from northern and western Germany. EqPV-H prevalence reached 7% and 35% (EqPV-H DNA positive detection and seroconversion, respectively). This study concerned mostly Thoroughbred brood mares, which represented 97% of the analyzed cohort. Concerning Thoroughbred stallions, Li et al. [14] identified a new equine papillomavirus (EcPV9) in the semen from an Australian Thoroughbred stallion suffering from a genital wart. The clinical significance of this new equine papillomavirus remained to be determined and will require further investigation. A similar question was raised by Nemoto et al. [15], who reported the first detection of equine coronavirus (ECoV) in Irish equids suffering from diarrhea. At five occasions, ECoV RNA was detected in feces from more than 400 equids with enteric diseases. However, the association with disease remains to be substantiated. While ECoV prevalence in Irish equids was 1.2% when measured by rRT-PCR in feces samples, evidence of ECoV infection was significantly higher when measured by serology in 984 serum samples from Dutch horses, 100 serums from Icelandic horses, and 27 paired serum samples from an ECoV outbreak in the USA. Zhao et al. [16] developed and validated an S1-protein-based ELISA for this purpose. Seroprevalence ranged from 26% in young horses to nearly 83% in adults. The authors highlighted the potential use of this ELISA as a diagnostic test to confirm ECoV outbreaks, as a complement to feces samples analysis by qRT-PCR. The study from Back et al. [17] shed some light on the potential role of equine rhinitis A virus (ERAV) infection in poor performance. This longitudinal study, which involved 30 Thoroughbred racehorses, significantly associated seroconversion to ERAV and subsequent failure to attend races. However, similarly to EqPV-H and ECoV infections previously reported in this Special Issue, a direct association of ERAV infection with clinical signs of disease could not be confirmed in this study.

Finally, Fatima et al. [18] investigated the antiviral activity of the equine interferon-mediated host factors myxovirus (Mx) protein (eqMx1) against a range of influenza A viruses (IAVs). The authors highlight the potential protective role of eqMx1, which primarily targets the virus nucleoprotein (NP), against the transmission of new IAVs in horses (i.e., eqMx1 could only inhibit the polymerase activity of IAVs of avian and human origin but remained inactive against the equine IAVs tested). Introduction of a new IAV in the equine population is considered a rare event. In 1989, an equine influenza epizootic was reported in the Jilin and Heilongjiang provinces of northeastern China, with up to 20% mortality, which is quite high when compared with conventional equine influenza outbreaks. The IAV strain representative of this outbreak (i.e., A/equine/Jilin/1/1989) was closely related to an avian H3N8 IAV [19]. The authors show that the IAV strain A/equine/Jilin/1/1989 bears two adaptive NP mutations that confer resistance to eqMx1. To date, equine influenza virus remains one of the most important respiratory pathogens of horses worldwide, with a potential damaging impact on the equine industry, as clearly illustrated in 2007 in Australia and in 2019 in Europe [20,21].

We hope this Special Issue helps to highlight the diversity of equine viruses and their importance, in terms of welfare and/or economic impact, to equids and humans.
